# The Molecular Basis of Retinal Dystrophies in Pakistan

**DOI:** 10.3390/genes5010176

**Published:** 2014-03-11

**Authors:** Muhammad Imran Khan, Maleeha Azam, Muhammad Ajmal, Rob W. J. Collin, Anneke I. den Hollander, Frans P. M. Cremers, Raheel Qamar

**Affiliations:** 1 Department of Biosciences, Faculty of Science, COMSATS Institute of Information Technology, Islamabad 45600, Pakistan; E-Mails: MuhammadImran.Khan@radboudumc.nl (M.I.K.); malihazam@gmail.com (M.Az.); chmajmal@gmail.com (M.Aj.); 2 Department of Human Genetics, Radboud University Medical Center, Nijmegen 6500 HB, The Netherlands; E-Mails: Rob.Collin@radboudumc.nl (R.W.J.C.); Anneke.denHollander@radboudumc.nl (A.I.H.); 3 Radboud Institute for Molecular Life Sciences, Radboud University Medical Center, Nijmegen 6500 HB, The Netherlands; 4 Department of Ophthalmology, Radboud University Medical Center, Nijmegen 6525 EX, The Netherlands; 5 Al-Nafees Medical College & Hospital, Isra University, Islamabad 45600, Pakistan

**Keywords:** inherited retinal dystrophies, homozygosity mapping, genetic testing

## Abstract

The customary consanguineous nuptials in Pakistan underlie the frequent occurrence of autosomal recessive inherited disorders, including retinal dystrophy (RD). In many studies, homozygosity mapping has been shown to be successful in mapping susceptibility loci for autosomal recessive inherited disease. RDs are the most frequent cause of inherited blindness worldwide. To date there is no comprehensive genetic overview of different RDs in Pakistan. In this review, genetic data of syndromic and non-syndromic RD families from Pakistan has been collected. Out of the 132 genes known to be involved in non-syndromic RD, 35 different genes have been reported to be mutated in families of Pakistani origin. In the Pakistani RD families 90% of the mutations causing non-syndromic RD and all mutations causing syndromic forms of the disease have not been reported in other populations. Based on the current inventory of all Pakistani RD-associated gene defects, a cost-efficient allele-specific analysis of 11 RD-associated variants is proposed, which may capture up to 35% of the genetic causes of retinal dystrophy in Pakistan.

## 1. Introduction

Inherited retinal dystrophies (RD) belong to a group of clinically and genetically heterogeneous disorders [1]. The clinical sub-classification of this group of diseases is based on the nature of the disease (stationary or progressive), the inheritance pattern, and the dysfunctional part of the retina [2]. The disease is either congenital, occurring early in life, such as Leber congenital amaurosis (LCA; MIM# 204000), and congenital stationary night blindness (CSNB; MIM# 310500), or might have a later onset, such as in retinitis pigmentosa (RP; MIM# 268000), cone-rod dystrophy (CRD; MIM# 604116), and cone dystrophy (CD; MIM# 602093) [3]. In addition to disorders confined to the eye, there are syndromic forms of the disease in which retinal dystrophy is either among the primary clinical symptoms or might manifest at an advanced stage. The most common syndromic form of RD is Usher syndrome (USH; MIM# 276900), in which RP is associated with variable degrees of hearing loss and vestibular dysfunction [4]. Other types of syndromic RD include Bardet-Biedl syndrome (BBS; MIM# 209900), Senior-Loken syndrome (SLSN; MIM# 266900), Joubert syndrome (JBTS; MIM# 213300), and Meckel syndrome (MKS; MIM# 249000). All these syndromes exhibit severe clinical features in addition to retinal degeneration [5,6].

The estimated worldwide prevalence of RD is 1 in 3000 individuals [7]. RP is the most frequent phenotype among the RDs, affecting 1 in 4000 individuals [8,9]. In Pakistan the frequency of RD is not very well defined, but a hospital-based study estimated autosomal recessive RP to be the most prevalent [10]. In several developing countries, as opposed to Western countries, consanguinity has always been a major contributing factor in the high prevalence of autosomal recessive disorders [11]. In Pakistan more than 60% of marriages are consanguineous and among them about 80% are between first cousins [12]. Such consanguineous families are ideal for homozygosity based genetic mapping studies aimed at the identification of the underlying genetic defect [13,14].

As a result of several technological advances, 201 genes implicated in different forms of RD have been identified to date [15]. Among these genes, 132 are linked to non-syndromic forms of the disease with some genetic overlap between different classes [1,3,16]. In the developed countries, genetic testing using medium-to-high throughput genotyping methods are now being routinely used for proper disease diagnosis [17]. This has resulted in the establishment of many genotype-phenotype correlations [17,18,19]. In the last two decades, several studies have described the genetic causes of different retinal dystrophies in consanguineous Pakistani families. However, to date, there has been no comprehensive ophthalmogenetic overview of all forms of RD that have been identified in Pakistan. Therefore, this literature review provides an overview of all published genetic data of syndromic and non-syndromic RD that have been described for Pakistani families.

## 2. Experimental

A comprehensive literature review was performed for mutations and loci, which have been described previously for Pakistani individuals with syndromic and non-syndromic retinal diseases. The Retinal Network (RetNet) [15], National Centre for Biotechnology Information (NCBI) [20], Online Mendelian Inheritance in Man (OMIM) [21], The Human Gene Mutation Database (HGMD) [22], and published literature were used to search for the causative genes. In order to predict the pathogenicity of the reported missense mutations, *in silico* analysis including, polymorphism phenotyping (PolyPhen-2) [23], and sorting tolerant from intolerant (SIFT) [24] were performed. The frequency of these variants in the healthy population was checked via the exome variant server (EVS) [25].

## 3. Results

### 3.1. Overview of Molecular Genetic Studies in Non-Syndromic RD in Pakistan

**Figure 1:**
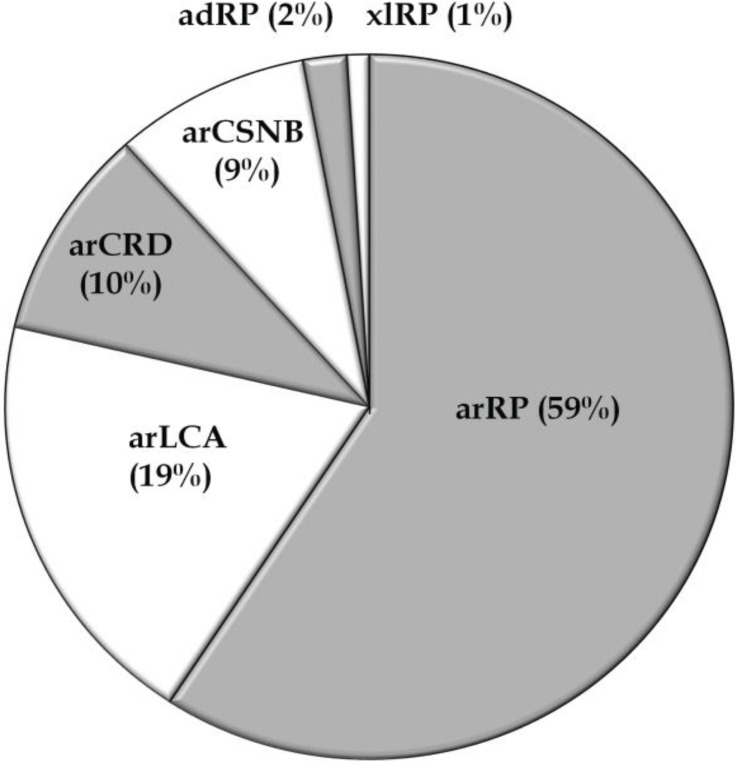
Distribution of non-syndromic Pakistani RD families according to their phenotypes. Ad, autosomal dominant; ar, autosomal recessive; CRD, cone-rod dystrophy; CSNB, congenital stationary night blindness; LCA, Leber congenital amaurosis; RP, retinitis pigmentosa; xl, X-linked.

**Figure 2:**
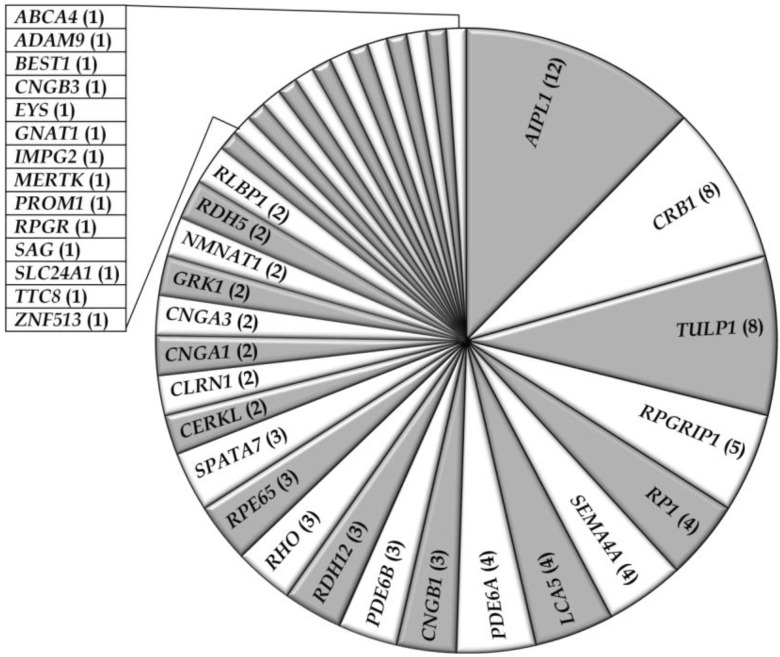
Occurrence of gene defects in non-syndromic RD families in Pakistan. Numbers of families with mutations in respective genes are indicated between parentheses.

Thus far, fifty-six studies have reported on the genetic causes of non-syndromic RD including arCRD, arCSNB, arLCA, and arRP in Pakistani persons, most of which belong to consanguineous families. The genetic data of a total of 466 Pakistani RD patients from 103 families (Table 1 and Table 2), have been described in the current review. Among these retinal phenotypes, arRP was found to be the most frequently occurring RD (59%), followed by arLCA (19%), arCRD (10%), and arCSNB (9%) (Table 1 and Table 2; Figure 1). Autosomal recessive inheritance seems to predominate in the RD families (96%) and only two autosomal dominant RP (adRP) families have been described (Table 1 and Table 2). Of these, one adRP family carries a mutation in *RHO* (MIM# 180380) [26], while in one family a frequent variant (c.2138G>A) in *SEMA4A* (MIM# 607292) has been described to cause adRP, however *in silico* prediction and exome variant server (EVS) frequency do not support the pathogenicity of the latter variant (Table 2) [27]. The compiled data demonstrate that out of the 132 genes known to be involved in non-syndromic RD, mutations in 36 different genes are causing disease in patients of Pakistani origin (Table 1; Figure 2), reflecting the genetic heterogeneity of the disease in this population. The most frequently mutated genes were *AIPL1* (MIM# 604392), *CRB1* (MIM# 604210), *TULP1* (MIM# 602280), *RPGRIP1* (MIM# 605446), *RP1* (MIM# 180100), *SEMA4A*, *LCA5* (MIM# 611408), and *PDE6A* (MIM# 180071) (Figure 2). Most of the reported mutations, and those identified in the current cohort, were novel to this population except for mutations in *ABCA4* (MIM# 601691), *CRB1*, *CERKL* (MIM# 608381), *RPE65* (MIM# 180069), *RPGR* (MIM# 312610), and *SPATA7* (MIM# 609868), which were initially identified in persons of different ethnicity (Table 1). As expected, all the reported disease associated alleles are rare variants and *in silico* analysis predicted these variants to have a deleterious effect on protein function (Table S1).

**Table 1:** Mutations identified in Pakistani patients with non-syndromic retinal dystrophies.

Gene	RefSeq Id	Nucleotide variant	Protein variant	Phenotype	# Families	# Patients	References
*ABCA4*	NM_000350.2	c.6658C>T	p.(Gln2220*)	arRP	1	6	[28,29]
*ADAM9*	NM_003816.2	c.766C>T	p.(Arg256*)	arCRD	1	4	[30]
*AIPL1* ^‡^	NM_201253.2	c.116C>A	p.(Thr39Asp)	arLCA	1	6	[31]
*AIPL1* ^‡^	NM_014336.3	c.834G>A	p.(Trp278*)	EORP	11	25	[29,31,32,33,34]
*BEST1* ^‡^	NM_001139443.1	c.418C>G	p.(Leu140Val)	arRP	1	4	[35]
*CERKL*	NM_001030311.2	c.316C>A	p.(Arg106Ser)	arRP	1	3	[36]
*CERKL*	NM_001030311.2	c.847C>T	p.(Arg283*)	arRP	1	6	[29,37,38]
*CLRN1* ^†^	NM_001195794.1	c.92C>T	p.(Pro31Leu)	arRP	1	6	[39]
*CLRN1* ^†^	NM_001195794.1	c.461T>G	p.(Leu154Trp)	arRP	1	6	[39]
*CNGA1*	NM_00142564.1	c.626_627del	p.(Ile209Serfs*26)	arRP	1	7	[40]
*CNGA1*	NM_00142564.1	c.1298G>A	P.(Gly433Asp)	arRP	1	3	[41]
*CNGA3*	NM_001298.2	c.822G>T	p.(Arg274Ser)	arCRD (ACHM)	1	4	[42]
*CNGA3*	NM_001298.2	c.827A>G	p.(Asn276Ser)	arCRD (ACHM)	1	6	[43]
*CNGB1*	NM_001297.4	c.412-1G>A	p.(?)	arRP	1	10	[44]
*CNGB1*	NM_001297.4	c.2284C>T	p.(Arg762Cys)	arRP	1	5	[44]
*CNGB1*	NM_001297.4	c.2493-2A>G	p.(?)	arRP	1	10	[41]
*CNGB3*	NM_019098.4	c.1825del	p.(Val609Trpfs*9)	arCRD (ACHM)	1	2	[42]
*CRB1*	NM_201253.2	c.107C>G	p.(Ser36*)	arLCA	1	10	[33]
*CRB1*	NM_201253.2	c.2234C>T	p.(Thr745Met)	arRP	1	2	[41,45]
*CRB1*	NM_201253.2	c.2536G>A	p.(Gly846Arg)	arRP	1	6	[31]
*CRB1*	NM_201253.2	c.3101T>C	p.(Leu989Thr)	arLCA	1	8	[31]
*CRB1*	NM_201253.2	c.3296C>A	p.(Thr1099Lys)	arRP	1	9	[44]
*CRB1*	NM_201253.2	c.3343_3352del	p.(Gly1115Ilefs*23)	arRP	1	9	[46]
*CRB1*	NM_201253.2	c.3347T>C	p.(Leu1071Pro)	arRP	1	7	[31]
*CRB1*	NM_201253.2	c.3962G>C	p.(Cys1321Ser)	arRP	1	5	[46]
*EYS*	NM_001142800.1	c.8299G>T	p.(Asp2767Tyr)	arRP	1	7	[47]
*GNAT1*	NM_144499.2	c.386A>G	p.(Asp129Gly)	arCSNB	1	1	[48]
*GRK1*	NM_ 002929	c.614C>A	p.(Ser205*)	arCSNB (Oguchi)	1	9	[49]
*GRK1*	NM_ 002929	c.827+623_883del	p.(?)	arCSNB (Oguchi)	1	3	[50]
*IMPG2* ^‡^	NM_016247.3	c.1680T>A	p.(Tyr560*)	arRP	1	2	[51]
*LCA5* ^‡^	NM_181714.3	c.643del	p.(Leu215Tyrfs*11)	arLCA	1	4	[52]
*LCA5* ^‡^	NM_181714.3	c.1151del	p.(Pro384Glnfs*17)	arLCA	3	13	[33,53]
*MERTK*	NM_00634.2	c.718G>T	p.(Glu240*)	arRP	1	4	[54]
*NMNAT1* ^‡^	NM_022787.3	c.25G>A	p.(Val9Met)	arLCA	1	5	[55]
*NMNAT1* ^‡^	NM_022787.3	c.838T>C	p.*280Glnext*16	arLCA	1	8	[56]
*PDE6A*	NM_000440.2	c.889C>T	p.(Gly297Ser)	arRP	1	4	[57]
*PDE6A*	NM_000440.2	c.1264-2A>G	p.(?)	arRP	1	5	[57]
*PDE6A*	NM_000440.2	c.1630C>T	p.(Arg544Trp)	arRP	1	3	[29]
*PDE6A*	NM_000440.2	c.2218_2219insT	p.(Ala740Valfs*2)	arRP	1	3	[57]
*PDE6B*	NM_000283.3	c.1160C>T	p.(Pro387Leu)	arRP	1	6	[58]
*PDE6B*	NM_000283.3	c.1655G>A	p.(Arg552Gln)	arRP	1	9	[58]
*PDE6B*	NM_000283.3	c.1722+1G>A	p.(?)	arRP	1	4	[44]
*PROM1*	NM_006017.2	c.1726C>T	p.(Gln576*)	arRP	1	6	[59]
*RDH12*	NM_152443.2	c.506G>A	p.(Arg169Gln)	arLCA/EORD	2	2	[60]
*RDH12*	NM_152443.2	c.619A>G	p.(Asn207Asp)	arLCA/EORD	1	1	[60]
*RDH5*	NM_001199771.1	c.758T>G	p.(Met253Arg)	arCSNB (FA)	1	6	[61]
*RDH5*	NM_001199771.1	c.913_917del	p.(Val305Hisfs*29)	arCSNB (FA)	1	2	[61]
*RHO*	NM_000539.3	c.448G>A	p.(Glu150Lys)	arRP	2	6	[62]
*RHO*	NM_000539.3	c.1045T>G	p.(*349Gluext*52)	adRP	1	8	[26]
*RLBP1*	NM_000326.4	c.346G>C	p.(Gly116Arg)	FA	1	4	[63]
*RLBP1*	NM_000326.4	c.466C>T	p.(Arg156*)	FA	1	6	[63]
*RP1*	NM_006269.1	c.1458_1461dup	p.(Glu488*)	arRP	2	9	[64,65]
*RP1*	NM_006269.1	c.4555del	p.(Arg1519Glufs*2)	arRP	1	5	[65]
*RP1*	NM_006269.1	c.5252del	p.(Asn1751Ilefs*4)	arRP	1	4	[65]
*RPE65*	NM_000329.2	c.131G>A	p.(Arg44Gln)	EORP	1	3	[41,66,67]
*RPE65*	NM_000329.2	c.361del	p.(Ser121Leufs*6)	EORP	1	4	[41,67]
*RPE65*	NM_000329.2	c.751G>T	p.(Val251Phe)	arLCA	1	6	[33]
*RPGR*	NM_001034853.1	c.2426_2427del	p.(Glu809Glyfs*25)	xlRP	1	8	[41,68]
*RPGRIP1*	NM_020366.3	c.587+1G>C	p.(?)	arLCA	1	1	[33]
*RPGRIP1*	NM_020366.3	c.1180C>T	p.(Gln394*)	arLCA	1	1	[33]
*RPGRIP1*	NM_020366.3	c.2480G>T	p.(Arg827Leu)	arCRD, arLCA	2	9	[33,69]
*RPGRIP1*	NM_020366.3	c.3620T>G	p.(Leu1207*)	arLCA	1	1	[33]
*SAG*	NM_000541.4	c.916G>T	p.(Glu306*)	arCSNB	1	1	[70]
*SEMA4A* ^‡^	NM_022367.3	c.1033G>C	p.(Asp345His)	arCRD, arRP	4	4	[27]
*SEMA4A* ^‡^	NM_022367.3	c.1049T>G	p.(Phe350Cys)				
*SLC24A1* ^‡^	NM_004727.2	c.1613_1614del	p.(Phe538Cysfs*23)	arCSNB	1	5	[71]
*SPATA7*	NM_018418.4	c.253C>T	p.(Arg85*)	arLCA/arRD	2	3	[72]
*SPATA7*	NM_018418.4	c.960dup	p.(Pro321Thrfs*6)	arLCA/arRD	1	6	[72,73]
*TTC8* ^†^	NM_144596.2	c.115-2A>G	p.(?)	arRP	1	4	[74]
*TULP1*	NM_003322.3	c.1138A>G	p.(Thr380Ala)	arRP	3	34	[33,75,76]
*TULP1*	NM_003322.3	c.1445G>A	p.(Arg482Gln)	arRP	1	8	[75]
*TULP1*	NM_003322.3	c.1466A>G	p.(Lys489Arg)	arRP	4	19	[41,76,77]
*ZNF513*	NM_144631.5	c.1015T>C	p.(Cys339Arg)	arRP	1	4	[78,79]

ACHM, achromatopsia; ad, autosomal dominant; ar, autosomal recessive; CSNB, congenital stationary night blindness; CRD, cone rod dystrophy; EORD, early onset retinal dystrophy; EORP, early onset RP; FA, fundus albipunctatus; LCA, Leber congenital amaurosis; RD, retinal dystrophy; RefSeq Id, reference sequence identifier; RP, retinitis pigmentosa; xlRP, X-linked RP; ^‡^ novel gene identification; ^†^ novel phenotype association.

Out of the 47 non-synonymous variants identified in Pakistani non-syndromic RD families (Table 1) three variants (*SEMA4A*, c.2138G>A; *RP1*, c.1118C>T; *RPGRIP1*, c.1639G>T), are reported as single nucleotide polymorphisms (SNP) with high frequencies in the EVS (Table 2) [27,64,69]. In addition, SIFT also predicts these changes to be tolerated while except for the *RPGRIP1* variant, the other two are considered to be benign by PolyPhen-2 (Table 2). Therefore, these variants could be segregating with the disease in the family by chance and the causative mutation may reside in another gene.

**Table 2:** Common variants reported as mutations in Pakistani patients with non-syndromic retinal dystrophies and their *in silico* pathogenicity prediction.

Gene	RefSeq Id	Nucleotide variant	Protein variant	Phenotype	# Families	# Patients	Ref.	phyloP	Grantham distance	PolyPhen	SIFT	EVS
*RP1*	NM_006269.1	c.1118C>T	p.(Thr373Ile)	arRP	2	11	[64]	0.61	89	Benign (0.01)	Tolerated (0.50)	T = 152; C = 12,854 (rs77775126)
*RPGRIP1*	NM_020366.3	c.1639G>T	p.(Ala547Ser)	arCRD	3	12	[69]	0.29	99	Probably damaging (1.00)	Tolerated (0.49)	T = 2,792; G = 9,214 (rs10151259)
*SEMA4A*	NM_022367.3	c.2138G>A	p.(Arg713Gln)	adRP	1	4	[27]	1.25	43	Benign (0.23)	Tolerated (0.43)	A = 451; G = 12,555 (rs41265017)

Ad, autosomal dominant; ar, autosomal recessive; CRD, cone-rod dystrophy; EVS, exome variant server; PolyPhen, polymorphism phenotyping; RefSeq Id, reference sequence identifier; RP, retinitis pigmentosa; SIFT, sorting tolerant from intolerant.

### 3.2. Overview of Molecular Genetic Studies in Syndromic RDs in Pakistan

**Figure 3:**
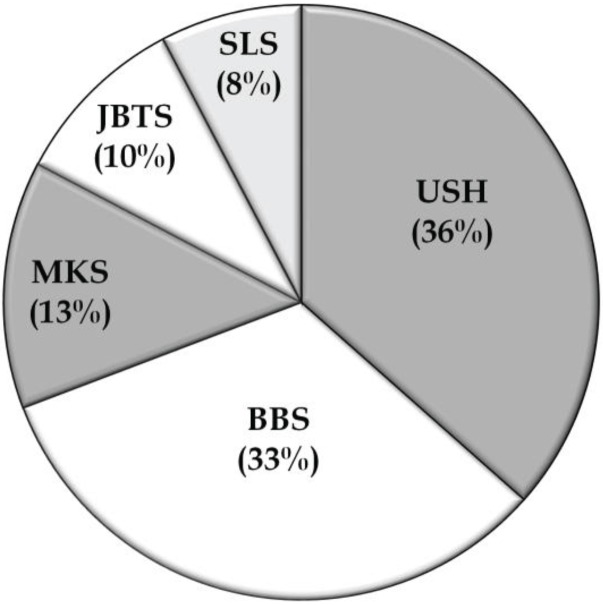
Prevalences of syndromic RD phenotypes. BBS, Bardet-Biedl syndrome; JBTS, Joubert syndrome; MKS, Meckel syndrome; SLS, Senior-Loken syndrome; USH, Usher syndrome.

**Figure 4:**
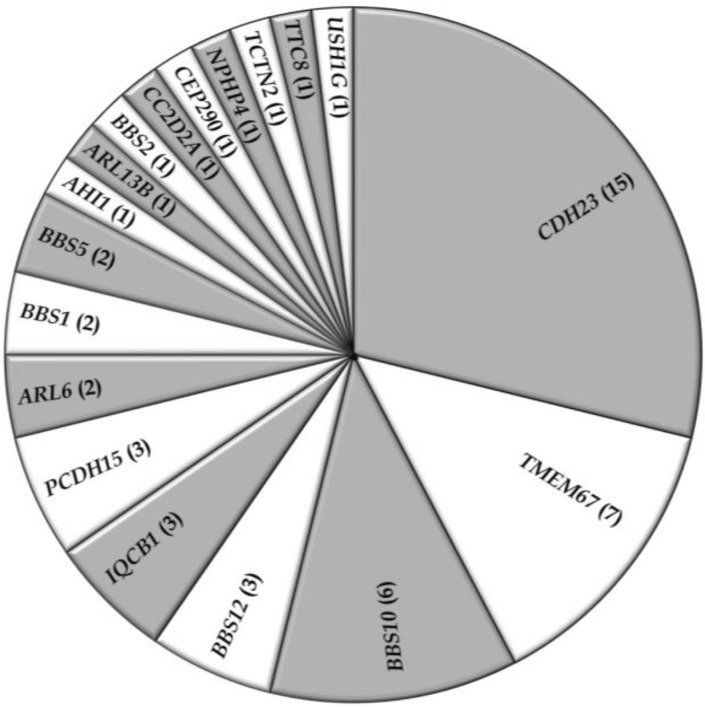
Occurrence of gene defects in syndromic RD families in Pakistan. Numbers of families with mutations in respective genes are indicated between parentheses.

In addition to the non-syndromic families, data of 52 syndromic RD families with a total of 139 affected individuals were collected from 22 studies. Usher syndrome represented about 36% of the families in this group, whereas BBS (33%), MKS (13%), JBTS (10%), and SLSN (8%), accounted for the other families (Table 3; Figure 3). The most commonly mutated gene associated with syndromic RD in the Pakistani population was cadherin 23 (*CDH23*; MIM# 605516), which has been reported to be mutated in persons with Usher type 1, followed by *TMEM67* (MIM# 609884), the gene mutated in persons with autosomal recessive MKS (Table 3; Figure 4). As expected for the syndromic mutations, all the reported disease associated alleles are rare variants and *in silico* analysis predicted these variants to have a deleterious effect on protein function (Table S2).

## 4. Discussion

The Pakistani population is known for its high rate of consanguinity (>60%), but it is still remarkable that 97% of the families with inherited RDs had an autosomal recessive mode of inheritance. It is, therefore, not surprising that Pakistani families have been instrumental in pinpointing a number of the underlying gene defects through homozygosity mapping [80,81]. Genetic studies of Pakistani families with RD have previously facilitated the identification of eleven novel RD genes, *i.e.*, *AIPL1* [34], *BEST1* [35], *CC2D2A* (MIM# 612013) [82], *CDH23* (MIM# 605516) [83], *IMPG2* (MIM# 607056) [51], *LCA5* (MIM# 611408) [53], *NMNAT1* (MIM:608700) [55,56], *ZNF513* (MIM# 613598) [78], *PCDH15* (MIM# 605514) [84], *SEMA4A* [27], and *SLC24A1* (MIM# 603617) [71]. In addition, mutations in *CLRN1* (MIM# 606397) and *TTC8* (MIM# 608132), which had been previously implicated in the syndromic retinal phenotypes USH3 (MIM# 276902), and BBS (MIM# 209900), respectively, were found to cause non-syndromic arRP [39,74]. Mutations in *RP1*, which had previously been shown to be involved in adRP, were found to segregate in a recessive manner in 3 Pakistani families [64]. In addition to the novel genes identified in the affected Pakistani families, five novel RD loci including three non-syndromic, *i.e.*, CORD8 (MIM# 605549), [85], RP29 (MIM# 612165), [86], and RP32 [87], and two syndromic, *i.e.*, USH1H (MIM# 612632), [88], and USH1K [89], have also been identified in Pakistani families.

**Table 3:** Mutations identified in Pakistani patients with syndromic retinal dystrophies.

Gene	RefSeq Id	Nucleotide variant	Protein variant	Phenotype	# Families	# Patients	References
*AHI1*	NM_017651.4	c.2370dup	p.(Lys791*)	arJBTS	1	2	[90]
*ARL6*	NM_032146.3	c.281T>C	p.(Ile94Thr)	arBBS	1	5	[91]
*ARL6*	NM_032146.3	c.123+1119del	p.(?)	arBBS	1	1	[92]
*ARL13B*	NM_182896.2	c.236G>A	p.(Arg79Gln)	arJBTS	1	3	[93]
*BBS1*	NM_02464.9.4	c.47+1G>T	p.(?)	arBBS	1	2	[94]
*BBS1*	NM_02464.9.4	c.442G>A	p.(Asp148Asn)	arBBS	1	2	[94]
*BBS2*	NM_031885.3	c.1237C>T	p.(Arg413*)	arBBS	1	1	[95]
*BBS5*	NM_152384.2	c.2T>A	p.(Met1Lys)	arBBS	2	2	[95]
*BBS10*	NM_024685.3	c.271dup	p.(Cys91Leufs*5)	arBBS	2	4	[96]
*BBS10*	NM_024685.3	c.1075C>T	p.(Gln359*)	arBBS	1	7	[91]
*BBS10*	NM_024685.3	c.1091del	p.(Asn364Thrfs*5)	arBBS	1	1	[96]
*BBS10*	NM_024685.3	c.1958_1967del	p.(Ser653Ilefs*4)	arBBS	1	2	[97]
*BBS10*	NM_024685.3	c.2121dup	p.(Lys708*)	arBBS	1	1	[96]
*BBS12*	NM_152618.2	c.1589T>C	p.(Leu530Pro)	arBBS	2	2	[95]
*BBS12*	NM_152618.2	c.2102C>A	p.(Ser701*)	arBBS	1	3	[98]
*CC2D2A* ^‡^	NM_001080522.2	c.2003+1G>C	p.(?)	arJBTS	1	5	[82]
*CDH23* ^‡^	NM_022124.5	c.1114C>T	p.(Gln372*)	arUSH1	1	3	[83]
*CDH23*	NM_022124.5	c.2587+1G>A	p.(?)	arUSH1	1	4	[99]
*CDH23*	NI	NI	p.(Arg1305*)	arUSH1	1	4	[99]
*CDH23* ^‡^	NM_022124.5	c.3106_3106+11delinsTGGT	p.(Gly1036delinsTrpCys)	arUSH1	1	5	[83]
*CDH23* ^‡^	NM_022124.5	c.6050-9G>A	p.(?)	arUSH1	4	13	[83]
*CDH23* ^‡^	NM_022124.5	c.6050-1G>C	p.(?)	arUSH1	1	6	[83]
*CDH23* ^‡^	NM_022124.5	c.6054_6074del	p.(Val2019_Val2025del)	arUSH1	1	3	[83]
CDH23 ^‡^	NM_022124.5	c.6845del	p.(Asn2282Thrfs*91)	arUSH1	1	3	[83]
*CDH23* ^‡^	NM_022124.5	c.7198C>T	p.(Pro2400Ser)	arUSH1	1	4	[83]
*CDH23* ^‡^	NM_022124.5	c.8150A>G	p.(Asp2717Gly)	arUSH1	1	3	[83]
CDH23 ^‡^	NM_022124.5	c.8208_8209del	p.(Val2737Alafs*2)	arUSH1	2	11	[83]
*CEP290*	NM_025114.3	c.5668G>T	p.(Gly1890*)	arJBTS	1	1	[100,83]
*IQCB1*	NM_001023570.2	c.488-1G>A	p.(?)	arSLSN	1	1	[41,102]
*IQCB1*	NM_001023570.2	c.1465C>T	p.(Arg489*)	arSLSN	1	1	[102]
*IQCB1*	NM_001023570.2	c.1796T>G	p.(*599Serext*2)	arSLSN	1	1	[102]
*NPHP4*	NM_015102.3	c.3272dup	p.(Ser1092Valfs*11)	arSLSN	1	1	[102]
*PCDH15* ^‡^	NM_001142763.1	c.7C>T	p.(Arg3*)	arUSH1	1	5	[84]
*PCDH15* ^‡^	NM_001142763.1	c.1927C>T	p.(Arg643*)	arUSH1	1	3	[103]
*PCDH15* ^‡^	NM_001142763.1	c.3389-2A>G	p.(?)	arUSH1	1	3	[84]
*TCTN2*	NM_024809.3	c.1873C>T	p.(Gln625*)	arJBTS	1	4	[104]
*TMEM67*	NM_153704.5	c.647del	p.(Val217Leufs*5)	arMKS	1	2	[105]
*TMEM67*	NM_153704.5	c.715-2A>G	p.(?)	arMKS	1	1	[105]
*TMEM67*	NM_153704.5	c.1127A>C	p.(Gln376Pro)	arMKS	2	2	[105]
*TMEM67*	NM_153704.5	c.1575+1G>A	p.(?)	arMKS	3	5	[105]
*TTC8*	NM_144596.2	c.1049+2_1049+4del	p.(?)	arBBS	1	3	[106]
*USH1G*	NM_173477.2	c.163_164+13del	p.(Gly56*)	arUSH1	1	4	[107]

Ar, autosomal recessive; BBS, Bardet-Biedl syndrome; JBTS, Joubert syndrome; MKS, Meckel syndrome; NI, not indicated; RefSeq Id, reference sequence identifier; SLSN, Senior-Loken syndrome; USH1, Usher syndrome type 1; ^‡^ novel gene identification; ^†^ novel phenotype association.

In the 103 non-syndromic Pakistani RD families described so far, mutations were most frequently found in *AIPL1*, *CRB1*, *TULP1*, *RPGRIP1*, *RP1*, *SEMA4A*, *LCA5*, and *PDE6A* (Table 1; Figure 2). A direct comparison with other RD populations is difficult as comprehensive studies of this kind are rare. In a recent study of Abu-Safieh *et al*. (2012) comprising 150 Saudi Arabian RD families, similar results were observed as *RP1*, *TULP1*, *RPGRIP1*, and *CRB1* were found to be the most frequently mutated genes [108].

A worldwide general literature study revealed arRP-associated mutations distributed in *USH2A* (12%; MIM# 276901), *ABCA4* (8%), *PDE6B* (7%; MIM# 180072), *CNGB1* (6%), and *PDE6A* (5%; MIM# 180071) [109]. In a more recent study of 230 Dutch persons with isolated or arRP [110], the most frequently mutated genes were *EYS* (11%; MIM# 602772), and *CRB1* (11%) followed by *USH2A* (10%), *ABCA4* (9%), and *PDE6B* (7%). As opposed to these studies the absence of *USH2A* variants in individuals of Pakistani origin is probably due to the fact that the most frequent arRP-associated variant, c.2299del;p.(E767fs), is almost invariably found in compound heterozygous states with second mutations that are considered to be mild [111], precluding their detection in a homozygosity mapping approach. Other differences can only be attributed to divergent genetic backgrounds of these populations [112,113].

Although 113/118 variants listed in Table 1 and Table 3 have only been identified in Pakistani patients, seven variants (*SEMA4A*, p.(Asp345His) and p.(Phe350Cys); *TULP1*, p.(Thr380Ala); *LCA5*, p.(Pro384Glnfs*17); *RPGRIP1*, p.(Arg827Leu); *TMEM67*, c.1575+1G>A and p.(Gln37Pro)), are more frequent than others, and therefore they seem to be population-specific. The six most frequent variants, p.(Trp278*) in *AIPL1*, p.(Lys489Arg) and p.(Thr380Ala) in *TULP1*, p.(Asp345His) and p.(Phe350Cys) in *SEMA4A*, p.(Pro384Glnfs*17) in *LCA5* (Table 1), explain about 25% of the non-syndromic Pakistani RD families. The p.Trp278* variant has been identified as the most frequent *AIPL1* variant worldwide in many LCA studies [114,115], suggesting that this variant is relatively old. The six frequent variants mentioned above, together with five other variants in *RDH12* (MIM# 608830), p.(Arg169Gln); *RHO*, p.(Glu150Lys); *RP1*, p.(Glu488*), *RPGRIP1*, p.(Arg827Leu), and *SPATA7*, p.(Arg85*), account for approximately 34% (35/103) of all non-syndromic RD families from Pakistan. A cost-effective initial genetic screening of Pakistani persons with RD therefore could be to analyze these variants using Sanger sequencing. For example, 10 amplicons covers the most frequent variants mentioned above. Alternatively, a larger subset of variants can be captured by arrayed primer extension (APEX) analysis or other allele-specific genotyping methods [116,117,118,119].

Three of the 47 missense mutations (*RP1*: c.1118C>T, *RPGRIP1*: c.1639G>T, *SEMA4A*: c.2138G>A) reported to be associated with RD in Pakistani families are found at higher frequencies in EVS. *In silico* analysis also predict them likely to be non-pathogenic, therefore they should be considered as non-causative (Table 2) [27,64,69]. As these variants on their own are not sufficient to explain the phenotype in these six families (two, three and one with *RP1*, *RPGRIP1* and *SEMA4A* mutations, respectively) they must still be considered genetically unresolved.

Of all the non-syndromic and syndromic arRD families (*n* = 146), which are genetically resolved, compound heterozygous mutations were identified in only four non-syndromic RD families (4/146 = 2.7%). These compound heterozygous mutations were identified in *SEMA4A*. This finding on one hand favors the utility of homozygosity based gene identification strategies for Pakistani RD families. While on the other hand it also indicates that in a small but significant proportion of the families (~2/100), compound heterozygous mutations might be able to explain the phenotype. These mutations will certainly be overlooked if one only considers homozygosity mapping based approaches to pinpoint causative genetic defects.

## 5. Conclusions

This review provides a comprehensive overview of genetic causes of non-syndromic and syndromic retinal diseases in Pakistan, the results of which can be used to design a cost-effective screening platform for future genetic testing in Pakistan. For genetically unsolved non-syndromic RD cases, we propose a sequencing-based pre-screening genetic test in which 10 different amplicons capture the most frequent mutations described for Pakistani RD patients. In consanguineous families, homozygosity directed sequence analysis has demonstrated its potential to unravel genetic defect underlying recessive diseases.

## Supplementary Files

Supplementary File 1 Supplementary Information (PDF, 52 KB)

## References

[1] Berger W., Kloeckener-Gruissem B., Neidhardt J. (2010). The molecular basis of human retinal and vitreoretinal diseases. Prog. Retin. Eye Res..

[2] Moradi P., Moore A.T. (2007). Molecular genetics of infantile-onset retinal dystrophies. Eye.

[3] Den Hollander A.I., Black A., Bennett J., Cremers F.P.M. (2010). Lighting a candle in the dark: Advances in genetics and gene therapy of recessive retinal dystrophies. J. Clin. Invest..

[4] Reiners J., Nagel-Wolfrum K., Jurgens K., Marker T., Wolfrum U. (2006). Molecular basis of human usher syndrome: Deciphering the meshes of the usher protein network provides insights into the pathomechanisms of the usher disease. Exp. Eye Res..

[5] Hildebrandt F., Zhou W. (2007). Nephronophthisis-associated ciliopathies. J. Am. Soc. Nephrol..

[6] Hildebrandt F., Benzing T., Katsanis N. (2011). Ciliopathies. N. Engl. J. Med..

[7] Robson A.G., Michaelides M., Saihan Z., Bird A.C., Webster A.R., Moore A.T., Fitzke F.W., Holder G.E. (2008). Functional characteristics of patients with retinal dystrophy that manifest abnormal parafoveal annuli of high density fundus autofluorescence; a review and update. Doc. Ophthalmol..

[8] Jay M. (1982). On the heredity of retinitis pigmentosa. Br. J. Ophthalmol..

[9] Ayuso C., Millan J.M. (2010). Retinitis pigmentosa and allied conditions today: A paradigm of translational research. Genome Med..

[10] Adhi M.I., Ahmed J. (2002). Frequency and clinical presentation of retinal dystrophies—A hospital based study. Pak. J. Ophthalmol..

[11] Bittles A.H. (2005). Endogamy, consanguinity and community disease profiles. Community Genet..

[12] Bittles A. (2001). Consanguinity and its relevance to clinical genetics. Clin. Genet..

[13] Lander E.S., Botstein D. (1987). Homozygosity mapping: A way to map human recessive traits with the DNA of inbred children. Science.

[14] Woods C.G., Cox J., Springell K., Hampshire D.J., Mohamed M.D., McKibbin M., Stern R., Raymond F.L., Sandford R., Malik Sharif S. (2006). Quantification of homozygosity in consanguineous individuals with autosomal recessive disease. Am. J. Hum. Genet..

[15] Retinal Information Network. https://sph.uth.edu/retnet/.

[16] Estrada-Cuzcano A., Roepman R., Cremers F.P.M., den Hollander A.I., Mans D.A. (2012). Non-syndromic retinal ciliopathies: Translating gene discovery into therapy. Hum. Mol. Genet..

[17] Downs K., Zacks D.N., Caruso R., Karoukis A.J., Branham K., Yashar B.M., Haimann M.H., Trzupek K., Meltzer M., Blain D. (2007). Molecular testing for hereditary retinal disease as part of clinical care. Arch. Ophthalmol..

[18] Koenekoop R.K., Lopez I., den Hollander A.I., Allikmets R., Cremers F.P.M. (2007). Genetic testing for retinal dystrophies and dysfunctions: Benefits, dilemmas and solutions. Clin. Exp. Ophthalmol..

[19] Brooks B.P., Macdonald I.M., Tumminia S.J., Smaoui N., Blain D., Nezhuvingal A.A., Sieving P.A. (2008). National Ophthalmic Disease Genotyping, N. Genomics in the era of molecular ophthalmology: Reflections on the national ophthalmic disease genotyping network (eyegene). Arch. Ophthalmol..

[20] National Centre for Biotechnology information. http://www.ncbi.nlm.nih.gov/pubmed/.

[21] Online Mendelian Inheritance in Man. http://www.omim.org/.

[22] The Human Gene Mutation Database. http://www.hgmd.cf.ac.uk/ac/index.php/.

[23] Adzhubei I.A., Schmidt S., Peshkin L., Ramensky V.E., Gerasimova A., Bork P., Kondrashov A.S., Sunyaev S.R. (2010). A method and server for predicting damaging missense mutations. Nat. Methods.

[24] Kumar P., Henikoff S., Ng P.C. (2009). Predicting the effects of coding non-synonymous variants on protein function using the sift algorithm. Nat. Protoc..

[25] NHLBI GO Exome Sequencing Project (ESP). http://evs.gs.washington.edu/EVS/.

[26] Bessant D.A., Khaliq S., Hameed A., Anwar K., Payne A.M., Mehdi S.Q., Bhattacharya S.S. (1999). Severe autosomal dominant retinitis pigmentosa caused by a novel rhodopsin mutation (Ter349Glu). Mutations in brief no. 208. Online. Hum. Mutat..

[27] Abid A., Ismail M., Mehdi S.Q., Khaliq S. (2006). Identification of novel mutations in the SEMA4A gene associated with retinal degenerative diseases. J. Med. Genet..

[28] Maugeri A., Klevering B.J., Rohrschneider K., Blankenagel A., Brunner H.G., Deutman A.F., Hoyng C.B., Cremers F.P.M. (2000). Mutations in the ABCA4 (ABCR) gene are the major cause of autosomal recessive cone-rod dystrophy. Am. J. Hum. Genet..

[29] Khan M.I., Ajmal M., Micheal S., Azam M., Hussain A., Shahzad A., Venselaar H., Bokhari H., de Wijs I., Hoefsloot L. (2013). Homozygosity mapping identifies genetic defects in four consanguineous families with retinal dystrophy from pakistan. Clin. Genet..

[30] Parry D.A., Toomes C., Bida L., Danciger M., Towns K.V., McKibbin M., Jacobson S.G., Logan C.V., Ali M., Bond J. (2009). Loss of the metalloprotease ADAM9 leads to cone-rod dystrophy in humans and retinal degeneration in mice. Am. J. Hum. Genet..

[31] Khaliq S., Abid A., Hameed A., Anwar K., Mohyuddin A., Azmat Z., Shami S.A., Ismail M., Mehdi S.Q. (2003). Mutation screening of Pakistani families with congenital eye disorders. Exp. Eye Res..

[32] Damji K.F., Sohocki M.M., Khan R., Gupta S.K., Rahim M., Loyer M., Hussein N., Karim N., Ladak S.S., Jamal A. (2001). Leber’s congenital amaurosis with anterior keratoconus in pakistani families is caused by the Trp278X mutation in the AIPL1 gene on 17p. Can. J. Ophthalmol..

[33] McKibbin M., Ali M., Mohamed M.D., Booth A.P., Bishop F., Pal B., Springell K., Raashid Y., Jafri H., Inglehearn C.F. (2010). Genotype-phenotype correlation for leber congenital amaurosis in Northern Pakistan. Arch. Ophthalmol..

[34] Sohocki M.M., Bowne S.J., Sullivan L.S., Blackshaw S., Cepko C.L., Payne A.M., Bhattacharya S.S., Khaliq S., Mehdi S.Q., Birch D.G. (2000). Mutations in a new photoreceptor-pineal gene on 17p cause leber congenital amaurosis. Nat. Genet..

[35] Davidson A.E., Millar I.D., Urquhart J.E., Burgess-Mullan R., Shweikh Y., Parry N., O’Sullivan J., Maher G.J., McKibbin M., Downes S.M. (2009). Missense mutations in a retinal pigment epithelium protein, bestrophin-1, cause retinitis pigmentosa. Am. J. Hum. Genet..

[36] Ali M., Ramprasad V.L., Soumittra N., Mohamed M.D., Jafri H., Rashid Y., Danciger M., McKibbin M., Kumaramanickavel G., Inglehearn C.F. (2008). A missense mutation in the nuclear localization signal sequence of CERKL (p.R106S) causes autosomal recessive retinal degeneration. Mol. Vis..

[37] Littink K.W., Koenekoop R.K., van den Born L.I., Collin R.W.J., Moruz L., Veltman J.A., Roosing S., Zonneveld M.N., Omar A., Darvish M. (2010). Homozygosity mapping in patients with cone-rod dystrophy: Novel mutations and clinical characterizations. Invest. Ophthalmol. Vis. Sci..

[38] Avila-Fernandez A., Riveiro-Alvarez R., Vallespin E., Wilke R., Tapias I., Cantalapiedra D., Aguirre-Lamban J., Gimenez A., Trujillo-Tiebas M.J., Ayuso C. (2008). CERKL mutations and associated phenotypes in seven spanish families with autosomal recessive retinitis pigmentosa. Invest. Ophthalmol. Vis. Sci..

[39] Khan M.I., Kersten F.F., Azam M., Collin R.W.J., Hussain A., Shah S.T.A., Keunen J.E.E., Kremer H., Cremers F.P.M., Qamar R. (2011). CLRN1 mutations cause nonsyndromic retinitis pigmentosa. Ophthalmology.

[40] Zhang Q., Zulfiqar F., Riazuddin S.A., Xiao X., Ahmad Z., Riazuddin S., Hejtmancik J.F. (2004). Autosomal recessive retinitis pigmentosa in a Pakistani family mapped to CNGA1 with identification of a novel mutation. Mol. Vis..

[41] Ajmal M. (2014). Personal Communications.

[42] Azam M., Collin R.W.J., Shah S.T.A., Shah A.A., Khan M.I., Hussain A., Sadeque A., Strom T.M., Thiadens A.A.H.J., Roosing S. (2010). Novel CNGA3 and CNGB3 mutations in two Pakistani families with achromatopsia. Mol. Vis..

[43] Saqib M.A., Awan B.M., Sarfraz M., Khan M.N., Rashid S., Ansar M. (2011). Genetic analysis of four Pakistani families with achromatopsia and a novel S4 motif mutation of CNGA3. Jpn. J. Ophthalmol..

[44] Azam M., Collin R.W.J., Malik A., Khan M.I., Shah S.T.A., Shah A.A., Hussain A., Sadeque A., Arimadyo K., Ajmal M. (2011). Identification of novel mutations in pakistani families with autosomal recessive retinitis pigmentosa. Arch. Ophthalmol..

[45] Den Hollander A.I., ten Brink J.B., de Kok Y.J.M., van Soest S., van den Born L.I., van Driel M.A., van de Pol T.J.R., Payne A.M., Bhattacharya S.S., Kellner U. (1999). Mutations in a human homologue of Drosophila crumbs cause retinitis pigmentosa (RP12). Nat. Genet..

[46] Lotery A.J., Malik A., Shami S.A., Sindhi M., Chohan B., Maqbool C., Moore P.A., Denton M.J., Stone E.M. (2001). CRB1 mutations may result in retinitis pigmentosa without para-arteriolar RPE preservation. Ophthalmic Genet..

[47] Khan M.I., Collin R.W.J., Arimadyo K., Micheal S., Azam M., Qureshi N., Faradz S.M.H., den Hollander A.I., Qamar R., Cremers F.P.M. (2010). Missense mutations at homologous positions in the fourth and fifth laminin A G-like domains of eyes shut homolog cause autosomal recessive retinitis pigmentosa. Mol. Vis..

[48] Naeem M.A., Chavali V.R., Ali S., Iqbal M., Riazuddin S., Khan S.N., Husnain T., Sieving P.A., Ayyagari R., Hejtmancik J.F. (2012). GNAT1 associated with autosomal recessive congenital stationary night blindness. Invest. Ophthalmol. Vis. Sci..

[49] Azam M., Collin R.W.J., Khan M.I., Shah S.T.A., Qureshi N., Ajmal M., den Hollander A.I., Qamar R., Cremers F.P.M. (2009). A novel mutation in GRK1 causes oguchi disease in a consanguineous Pakistani family. Mol. Vis..

[50] Zhang Q., Zulfiqar F., Riazuddin S.A., Xiao X., Yasmeen A., Rogan P.K., Caruso R., Sieving P.A., Riazuddin S., Hejtmancik J.F. (2005). A variant form of Oguchi disease mapped to 13q34 associated with partial deletion of GRK1 gene. Mol. Vis..

[51] Bandah-Rozenfeld D., Collin R.W.J., Banin E., van den Born L.I., Coene K.L.M., Siemiatkowska A.M., Zelinger L., Khan M.I., Lefeber D.J., Erdinest I. (2010). Mutations in IMPG2, encoding interphotoreceptor matrix proteoglycan 2, cause autosomal-recessive retinitis pigmentosa. Am. J. Hum. Genet..

[52] Ahmad A., Daud S., Kakar N., Nurnberg G., Nurnberg P., Babar M.E., Thoenes M., Kubisch C., Ahmad J., Bolz H.J. (2011). Identification of a novel LCA5 mutation in a Pakistani family with Leber congenital amaurosis and cataracts. Mol. Vis..

[53] Den Hollander A.I., Koenekoop R.K., Mohamed M.D., Arts H.H., Boldt K., Towns K.V., Sedmak T., Beer M., Nagel-Wolfrum K., McKibbin M. (2007). Mutations in LCA5, encoding the ciliary protein lebercilin, cause leber congenital amaurosis. Nat. Genet..

[54] Shahzadi A., Riazuddin S.A., Ali S., Li D., Khan S.N., Husnain T., Akram J., Sieving P.A., Hejtmancik J.F., Riazuddin S. (2010). Nonsense mutation in MERTK causes autosomal recessive retinitis pigmentosa in a consanguineous Pakistani family. Br. J. Ophthalmol..

[55] Falk M.J., Zhang Q., Nakamaru-Ogiso E., Kannabiran C., Fonseca-Kelly Z., Chakarova C., Audo I., Mackay D.S., Zeitz C., Borman A.D. (2012). NMNAT1 mutations cause leber congenital amaurosis. Nat. Genet..

[56] Koenekoop R.K., Wang H., Majewski J., Wang X., Lopez I., Ren H., Chen Y., Li Y., Fishman G.A., Genead M. (2012). Mutations in NMNAT1 cause leber congenital amaurosis and identify a new disease pathway for retinal degeneration. Nat. Genet..

[57] Riazuddin S.A., Zulfiqar F., Zhang Q., Yao W., Li S., Jiao X., Shahzadi A., Amer M., Iqbal M., Hussnain T. (2006). Mutations in the gene encoding the alpha-subunit of rod phosphodiesterase in consanguineous Pakistani families. Mol. Vis..

[58] Ali S., Riazuddin S.A., Shahzadi A., Nasir I.A., Khan S.N., Husnain T., Akram J., Sieving P.A., Hejtmancik J.F., Riazuddin S. (2011). Mutations in the beta-subunit of rod phosphodiesterase identified in consanguineous Pakistani families with autosomal recessive retinitis pigmentosa. Mol. Vis..

[59] Zhang Q., Zulfiqar F., Xiao X., Riazuddin S.A., Ahmad Z., Caruso R., MacDonald I., Sieving P., Riazuddin S., Hejtmancik J.F. (2007). Severe retinitis pigmentosa mapped to 4p15 and associated with a novel mutation in the PROM1 gene. Hum. Genet..

[60] Mackay D.S., Dev Borman A., Moradi P., Henderson R.H., Li Z., Wright G.A., Waseem N., Gandra M., Thompson D.A., Bhattacharya S.S. (2011). RDH12 retinopathy: Novel mutations and phenotypic description. Mol. Vis..

[61] Ajmal M., Khan M.I., Neveling K., Khan Y.M., Ali S.H., Ahmed W., Iqbal M.S., Azam M., den Hollander A.I., Collin R.W.J. (2012). Novel mutations in RDH5 cause fundus albipunctatus in two consanguineous Pakistani families. Mol. Vis..

[62] Azam M., Khan M.I., Gal A., Hussain A., Shah S.T.A., Khan M.S., Sadeque A., Bokhari H., Collin R.W.J., Orth U. (2009). A homozygous p.Glu150Lys mutation in the opsin gene of two pakistani families with autosomal recessive retinitis pigmentosa. Mol. Vis..

[63] Naz S., Ali S., Riazuddin S.A., Farooq T., Butt N.H., Zafar A.U., Khan S.N., Husnain T., Macdonald I.M., Sieving P.A. (2011). Mutations in RLBP1 associated with fundus albipunctatus in consanguineous Pakistani families. Br. J. Ophthalmol..

[64] Khaliq S., Abid A., Ismail M., Hameed A., Mohyuddin A., Lall P., Aziz A., Anwar K., Mehdi S.Q. (2005). Novel association of RP1 gene mutations with autosomal recessive retinitis pigmentosa. J. Med. Genet..

[65] Riazuddin S.A., Zulfiqar F., Zhang Q., Sergeev Y.V., Qazi Z.A., Husnain T., Caruso R., Riazuddin S., Sieving P.A., Hejtmancik J.F. (2005). Autosomal recessive retinitis pigmentosa is associated with mutations in RP1 in three consanguineous Pakistani families. Invest. Ophthalmol. Vis. Sci..

[66] Simovich M.J., Miller B., Ezzeldin H., Kirkland B.T., McLeod G., Fulmer C., Nathans J., Jacobson S.G., Pittler S.J. (2001). Four novel mutations in the RPE65 gene in patients with Leber congenital amaurosis. Hum. Mutat..

[67] Coppieters F., de Baere E., Leroy B. (2011). Development of a next-generation sequencing platform for retinal dystrophies, with LCA and RP as proof of concept. Bull. Soc. Belg. Ophtalmol..

[68] Vervoort R., Lennon A., Bird A.C., Tulloch B., Axton R., Miano M.G., Meindl A., Meitinger T., Ciccodicola A., Wright A.F. (2000). Mutational hot spot within a new RPGR exon in X-linked retinitis pigmentosa. Nat. Genet..

[69] Hameed A., Abid A., Aziz A., Ismail M., Mehdi S.Q., Khaliq S. (2003). Evidence of rpgrip1 gene mutations associated with recessive cone-rod dystrophy. J. Med. Genet..

[70] Waheed N.K., Qavi A.H., Malik S.N., Maria M., Riaz M., Cremers F.P.M., Azam M., Qamar R. (2012). A nonsense mutation in S-antigen (p.Glu306*) causes Oguchi disease. Mol. Vis..

[71] Riazuddin S.A., Shahzadi A., Zeitz C., Ahmed Z.M., Ayyagari R., Chavali V.R., Ponferrada V.G., Audo I., Michiels C., Lancelot M.E. (2010). A mutation in SLC24A1 implicated in autosomal-recessive congenital stationary night blindness. Am. J. Hum. Genet..

[72] Mackay D.S., Ocaka L.A., Borman A.D., Sergouniotis P.I., Henderson R.H., Moradi P., Robson A.G., Thompson D.A., Webster A.R., Moore A.T. (2011). Screening of SPATA7 in patients with Leber congenital amaurosis and severe childhood-onset retinal dystrophy reveals disease-causing mutations. Invest. Ophthalmol. Vis. Sci..

[73] Wang H., den Hollander A.I., Moayedi Y., Abulimiti A., Li Y., Collin R.W.J., Hoyng C.B., Lopez I., Abboud E.B., Al-Rajhi A.A. (2009). Mutations in SPATA7 cause Leber congenital amaurosis and juvenile retinitis pigmentosa. Am. J. Hum. Genet..

[74] Riazuddin S.A., Iqbal M., Wang Y., Masuda T., Chen Y., Bowne S., Sullivan L.S., Waseem N.H., Bhattacharya S., Daiger S.P. (2010). A splice-site mutation in a retina-specific exon of BBS8 causes nonsyndromic retinitis pigmentosa. Am. J. Hum. Genet..

[75] Ajmal M., Khan M.I., Micheal S., Ahmed W., Shah A., Venselaar H., Bokhari H., Azam A., Waheed N.K., Collin R.W.J. (2012). Identification of recurrent and novel mutations in TULP1 in Pakistani families with early-onset retinitis pigmentosa. Mol. Vis..

[76] Iqbal M., Naeem M.A., Riazuddin S.A., Ali S., Farooq T., Qazi Z.A., Khan S.N., Husnain T., Riazuddin S., Sieving P.A. (2011). Association of pathogenic mutations in TULP1 with retinitis pigmentosa in consanguineous Pakistani families. Arch. Ophthalmol..

[77] Gu S., Lennon A., Li Y., Lorenz B., Fossarello M., North M., Gal A., Wright A. (1998). Tubby-like protein-1 mutations in autosomal recessive retinitis pigmentosa. Lancet.

[78] Li L., Nakaya N., Chavali V.R., Ma Z., Jiao X., Sieving P.A., Riazuddin S., Tomarev S.I., Ayyagari R., Riazuddin S.A. (2010). A mutation in ZNF513, a putative regulator of photoreceptor development, causes autosomal-recessive retinitis pigmentosa. Am. J. Hum. Genet..

[79] Naz S., Riazuddin S.A., Li L., Shahid M., Kousar S., Sieving P.A., Hejtmancik J.F., Riazuddin S. (2010). A novel locus for autosomal recessive retinitis pigmentosa in a consanguineous Pakistani family maps to chromosome 2p. Am. J. Ophthalmol..

[80] Rafiq M.A., Ansar M., Marshall C.R., Noor A., Shaheen N., Mowjoodi A., Khan M.A., Ali G., Amin-ud-Din M., Feuk L. (2010). Mapping of three novel loci for non-syndromic autosomal recessive mental retardation (NS-ARMR) in consanguineous families from pakistan. Clin. Genet..

[81] Kakar N., Goebel I., Daud S., Nurnberg G., Agha N., Ahmad A., Nurnberg P., Kubisch C., Ahmad J., Borck G. (2012). A homozygous splice site mutation in TRAPPC9 causes intellectual disability and microcephaly. Eur. J. Med. Genet..

[82] Noor A., Windpassinger C., Patel M., Stachowiak B., Mikhailov A., Azam M., Irfan M., Siddiqui Z.K., Naeem F., Paterson A.D. (2008). CC2D2A, encoding a coiled-coil and C2 domain protein, causes autosomal-recessive mental retardation with retinitis pigmentosa. Am. J. Hum. Genet..

[83] Schultz J.M., Bhatti R., Madeo A.C., Turriff A., Muskett J.A., Zalewski C.K., King K.A., Ahmed Z.M., Riazuddin S., Ahmad N. (2011). Allelic hierarchy of CDH23 mutations causing non-syndromic deafness DFNB12 or usher syndrome USH1D in compound heterozygotes. J. Med. Genet..

[84] Ahmed Z.M., Riazuddin S., Bernstein S.L., Ahmed Z., Khan S., Griffith A.J., Morell R.J., Friedman T.B., Wilcox E.R. (2001). Mutations of the protocadherin gene PCDH15 cause usher syndrome type 1f. Am. J. Hum. Genet..

[85] Ismail M., Abid A., Anwar K., Mehdi S.Q., Khaliq S. (2006). Refinement of the locus for autosomal recessive cone-rod dystrophy (CORD8) linked to chromosome 1q23-q24 in a pakistani family and exclusion of candidate genes. J. Hum. Genet..

[86] Hameed A., Khaliq S., Ismail M., Anwar K., Mehdi S.Q., Bessant D., Payne A.M., Bhattacharya S.S. (2001). A new locus for autosomal recessive RP (RP29) mapping to chromosome 4q32-q34 in a pakistani family. Invest. Ophthalmol. Vis. Sci..

[87] Zhang Q., Zulfiqar F., Xiao X., Riazuddin S.A., Ayyagari R., Sabar F., Caruso R., Sieving P.A., Riazuddin S., Hejtmancik J.F. (2005). Severe autosomal recessive retinitis pigmentosa maps to chromosome 1p13.3-p21.2 between D1S2896 and D1S457 but outside ABCA4. Hum. Genet..

[88] Ahmed Z.M., Riazuddin S., Khan S.N., Friedman P.L., Riazuddin S., Friedman T.B. (2009). USH1H, a novel locus for type I Usher syndrome, maps to chromosome 15q22-23. Clin. Genet..

[89] Jaworek T.J., Bhatti R., Latief N., Khan S.N., Riazuddin S., Ahmed Z.M. (2012). USH1K, a novel locus for type I Usher syndrome, maps to chromosome 10p11.21-q21.1. J. Hum. Genet..

[90] Utsch B., Sayer J.A., Attanasio M., Pereira R.R., Eccles M., Hennies H.C., Otto E.A., Hildebrandt F. (2006). Identification of the first AHI1 gene mutations in nephronophthisis-associated Joubert syndrome. Pediatr. Nephrol..

[91] Khan S., Ullah I., Irfanullah I., Touseef M., Basit S., Khan M.N., Ahmad W. (2013). Novel homozygous mutations in the genes ARL6 and BBS10 underlying Bardet-Biedl syndrome. Gene.

[92] Chen J., Smaoui N., Hammer M.B., Jiao X., Riazuddin S.A., Harper S., Katsanis N., Riazuddin S., Chaabouni H., Berson E.L. (2011). Molecular analysis of Bardet-Biedl syndrome families: Report of 21 novel mutations in 10 genes. Invest. Ophthalmol. Vis. Sci..

[93] Cantagrel V., Silhavy J.L., Bielas S.L., Swistun D., Marsh S.E., Bertrand J.Y., Audollent S., Attie-Bitach T., Holden K.R., Dobyns W.B. (2008). Mutations in the cilia gene ARL13B lead to the classical form of Joubert syndrome. Am. J. Hum. Genet..

[94] Ajmal M., Khan M.I., Neveling K., Tayyab A., Jaffar S., Sadeque A., Ayub H., Abbasi N.M., Riaz M., Micheal S. (2013). Exome sequencing identifies a novel and a recurrent BBS1 mutation in Pakistani families with Bardet-Biedl syndrome. Mol. Vis..

[95] Harville H.M., Held S., Diaz-Font A., Davis E.E., Diplas B.H., Lewis R.A., Borochowitz Z.U., Zhou W., Chaki M., Macdonald J. (2010). Identification of 11 novel mutations in eight BBS genes by high-resolution homozygosity mapping. J. Med. Genet..

[96] White D.R., Ganesh A., Nishimura D., Rattenberry E., Ahmed S., Smith U.M., Pasha S., Raeburn S., Trembath R.C., Rajab A. (2007). Autozygosity mapping of Bardet-Biedl syndrome to 12q21.2 and confirmation of FLJ23560 as BBS10. Eur. J. Hum. Genet..

[97] Agha Z., Iqbal Z., Azam M., Hoefsloot L.H., van Bokhoven H., Qamar R. (2013). A novel homozygous 10 nucleotide deletion in BBS10 causes Bardet-Biedl syndrome in a Pakistani family. Gene.

[98] Pawlik B., Mir A., Iqbal H., Li Y., Nurnberg G., Becker C., Qamar R., Nurnberg P., Wollnik B. (2010). A novel familial BBS12 mutation associated with a mild phenotype: Implications for clinical and molecular diagnostic strategies. Mol. Syndromol..

[99] Bork J.M., Peters L.M., Riazuddin S., Bernstein S.L., Ahmed Z.M., Ness S.L., Polomeno R., Ramesh A., Schloss M., Srisailpathy C.R. (2001). Usher syndrome 1D and nonsyndromic autosomal recessive deafness DFNB12 are caused by allelic mutations of the novel cadherin-like gene CDH23. Am. J. Hum. Genet..

[100] Otto E.A., Ramaswami G., Janssen S., Chaki M., Allen S.J., Zhou W., Airik R., Hurd T.W., Ghosh A.K., Wolf M.T. (2011). Mutation analysis of 18 nephronophthisis associated ciliopathy disease genes using a DNA pooling and next generation sequencing strategy. J. Med. Genet..

[101] Sayer J.A., Otto E.A., O’Toole J.F., Nurnberg G., Kennedy M.A., Becker C., Hennies H.C., Helou J., Attanasio M., Fausett B.V. (2006). The centrosomal protein nephrocystin-6 is mutated in joubert syndrome and activates transcription factor ATF4. Nat. Genet..

[102] Otto E.A., Helou J., Allen S.J., O’Toole J.F., Wise E.L., Ashraf S., Attanasio M., Zhou W., Wolf M.T.F., Hildebrandt F. (2008). Mutation analysis in nephronophthisis using a combined approach of homozygosity mapping, CEL I endonuclease cleavage, and direct sequencing. Hum. Mutat..

[103] Ahmed Z.M., Riazuddin S., Ahmad J., Bernstein S.L., Guo Y., Sabar M.F., Sieving P., Griffith A.J., Friedman T.B., Belyantseva I.A. (2003). PCDH15 is expressed in the neurosensory epithelium of the eye and ear and mutant alleles are responsible for both USH1F and DFNB23. Hum. Mol. Genet..

[104] Sang L., Miller J.J., Corbit K.C., Giles R.H., Brauer M.J., Otto E.A., Baye L.M., Wen X., Scales S.J., Kwong M. (2011). Mapping the NPHP-JBTS-MKS protein network reveals ciliopathy disease genes and pathways. Cell.

[105] Smith U.M., Consugar M., Tee L.J., McKee B.M., Maina E.N., Whelan S., Morgan N.V., Goranson E., Gissen P., Lilliquist S. (2006). The transmembrane protein meckelin (MKS3) is mutated in Meckel-Gruber syndrome and the wpk rat. Nat. Genet..

[106] Ansley S.J., Badano J.L., Blacque O.E., Hill J., Hoskins B.E., Leitch C.C., Kim J.C., Ross A.J., Eichers E.R., Teslovich T.M. (2003). Basal body dysfunction is a likely cause of pleiotropic Bardet-Biedl syndrome. Nature.

[107] Bashir R., Fatima A., Naz S. (2010). A frameshift mutation in SANS results in atypical Usher syndrome. Clin. Genet..

[108] Abu-Safieh L., Alrashed M., Anazi S., Alkuraya H., Khan A.O., Al-Owain M., Al-Zahrani J., Al-Abdi L., Hashem M., Al-Tarimi S. (2013). Autozygome-guided exome sequencing in retinal dystrophy patients reveals pathogenetic mutations and novel candidate disease genes. Genome Res..

[109] Hartong D.T., Berson E.L., Dryja T.P. (2006). Retinitis pigmentosa. Lancet.

[110] Neveling K., Collin R.W.J., Gilissen C., van Huet R.A., Visser L., Kwint M.P., Gijsen S.J., Zonneveld M.N., Wieskamp N., de Ligt J. (2012). Next-generation genetic testing for retinitis pigmentosa. Hum. Mutat..

[111] Seyedahmadi B.J., Rivolta C., Keene J.A., Berson E.L., Dryja T.P. (2004). Comprehensive screening of the USH2A gene in usher syndrome type II and non-syndromic recessive retinitis pigmentosa. Exp. Eye Res..

[112] Qamar R., Ayub Q., Mohyuddin A., Helgason A., Mazhar K., Mansoor A., Zerjal T., Tyler-Smith C., Mehdi S.Q. (2002). Y-chromosomal DNA variation in pakistan. Am. J. Hum. Genet..

[113] Collin R.W.J., van den Born L.I., Klevering B.J., de Castro-Miro M., Littink K.W., Arimadyo K., Azam M., Yazar V., Zonneveld M.N., Paun C.C. (2011). High-resolution homozygosity mapping is a powerful tool to detect novel mutations causative of autosomal recessive RP in the dutch population. Invest. Ophthalmol. Vis. Sci..

[114] Sohocki M.M., Perrault I., Leroy B.P., Payne A.M., Dharmaraj S., Bhattacharya S.S., Kaplan J., Maumenee I.H., Koenekoop R., Meire F.M. (2000). Prevalence of AIPL1 mutations in inherited retinal degenerative disease. Mol. Genet. Metab..

[115] Yzer S., Leroy B.P., de Baere E., de Ravel T.J., Zonneveld M.N., Voesenek K., Kellner U., Martinez Ciriano J.P., de Faber J.T.H.N., Rohrschneider K. (2006). Microarray-based mutation detection and phenotypic characterization of patients with leber congenital amaurosis. Invest. Ophthalmol. Vis. Sci..

[116] Muller J., Stoetzel C., Vincent M.C., Leitch C.C., Laurier V., Danse J.M., Helle S., Marion V., Bennouna-Greene V., Vicaire S. (2010). Identification of 28 novel mutations in the Bardet-Biedl syndrome genes: The burden of private mutations in an extensively heterogeneous disease. Hum. Genet..

[117] Kurg A., Tonisson N., Georgiou I., Shumaker J., Tollett J., Metspalu A. (2000). Arrayed primer extension: Solid-phase four-color DNA resequencing and mutation detection technology. Genet. Test..

[118] Jaakson K., Zernant J., Kulm M., Hutchinson A., Tonisson N., Hawlina M., Ravnic-Glavac M., Meltzer M., Caruso R., Testa F. (2003). Genotyping microarray (gene chip) for the ABCR (ABCA4) gene. Hum. Mutat..

[119] Avila-Fernandez A., Cantalapiedra D., Aller E., Vallespin E., guirre-Lamban J., Blanco-Kelly F., Corton M., Riveiro-Alvarez R., Allikmets R., Trujillo-Tiebas M.J. (2010). Mutation analysis of 272 spanish families affected by autosomal recessive retinitis pigmentosa using a genotyping microarray. Mol. Vis..

