# Supplementary material for: The Molecular Basis of Retinal Dystrophies in Pakistan
Source: Genes (Basel). 2014 Mar 11;5(1):176–95. doi: 10.3390/genes5010176 (PMC3978518; doi:10.3390/genes5010176)
Supplement: Supplementary File 1 — Supplementary Information (PDF, 52 KB) [file genes-05-00176-s001.pdf]

Supplementary Information

Table S1. *In silico* pathogenicity prediction of variants identified in Pakistani individuals with non-syndromic retinal dystrophies and their frequencies in normal individuals.

Gene	RefSeq Id	Nucleotide variant	Protein variant	PhyloP	Grantham distance	PolyPhen	SIFT	EVS
AIPL1	NM_201253.2	c.116C>A	p.(Thr39Asp)	3.60	65	Probably damaging (1.00)	Deleterious (0.01)	Absent
BEST1	NM_001139443.1	c.418C>G	p.(Leu140Val)	1.34	32	Probably damaging (1.00)	Deleterious (0.03)	Absent
CERKL	NM_001030311.2	c.316C>A	p.(Arg106Ser)	4.48	110	Probably damaging (1.00)	Tolerated (0.82)	Absent
CLRN1	NM_174878.2	c.92C>T	p.(Pro31Leu)	2.14	98	Probably damaging (0.98)	Deleterious (0.04)	Absent
CLRN1	NM_174878.2	c.461T>G	p.(Leu154Trp)	4.48	61	Probably damaging (1.00)	Deleterious (0.00)	Absent
CNGA1	NM_00142564.1	c.1298G>A	p.(Gly433Asp)	5.77	94	Probably damaging (1.00)	Deleterious (0.00)	Absent
CNGA3	NM_001298.2	c.822G>T	p.(Arg274Ser)	0.21	110	Probably damaging (1.00)	Deleterious (0.00)	Absent
CNGA3	NM_001298.2	c.827A>G	p.(Asn276Ser)	4.73	46	Probably damaging (0.99)	Deleterious (0.00)	Absent
CNGB1	NM_001297.4	c.2284C>T	p.(Arg762Cys)	1.58	180	Probably damaging (1.00)	Deleterious (0.00)	Absent
CRB1	NM_201253.2	c.2234C>T	p.(Thr745Met)	4.16	81	Probably damaging (1.00)	Deleterious (0.00)	T = 1; C = 13,005
CRB1	NM_201253.2	c.2536G>A	p.(Gly846Arg)	3.76	125	Probably damaging (1.00)	Deleterious (0.00)	Absent
CRB1	NM_201253.2	c.3101T>C	p.(Leu989Thr)	4.64	89	Probably damaging (0.95)	Deleterious (0.00)	Absent
CRB1	NM_201253.2	c.3296C>A	p.(Thr1099Lys)	2.95	78	Probably damaging (0.98)	Deleterious (0.00)	Absent
CRB1	NM_201253.2	c.3347T>C	p.(Leu1071Pro)	4.73	98	Probably damaging (1.00)	Deleterious (0.00)	Absent
CRB1	NM_201253.2	c.3962G>C	p.(Cys1321Ser)	4.32	112	Probably damaging (0.85)	Deleterious (0.00)	Absent
EYS	NM_001142800.1	c.8299G>T	p.(Asp2767Tyr)	2.71	160	Probably damaging (1.00)	Deleterious (0.01)	Absent
GNAT1	NM_144499.2	c.386A>G	p.(Asp129Gly)	4.64	94	Probably damaging (1.00)	Deleterious (0.00)	Absent
NMNAT1	NM_022787.3	c.25G>A	p.(Val9Met)	2.38	21	Probably damaging (0.98)	Deleterious (0.02)	Absent
PDE6A	NM_000440.2	c.889C>T	p.(Gly297Ser)	5.45	56	Probably damaging (1.00)	Tolerated (0.12)	Absent
PDE6A	NM_000440.2	c.1630C>T	p.(Arg544Trp)	1.98	101	Probably damaging (0.99)	Deleterious (0.02)	A = 1; G = 13,005
PDE6B	NM_000283.3	c.1160C>T	p.(Pro387Leu)	5.29	98	Probably damaging (1.00)	Deleterious (0.01)	Absent
PDE6B	NM_000283.3	c.1655G>A	p.(Arg552Gln)	4.56	43	Probably damaging (0.99)	Tolerated (0.06)	Absent
RDH12	NM_152443.2	c.506G>A	p.(Arg169Gln)	6.26	43	Probably damaging (1.00)	Deleterious (0.00)	Absent
RDH12	NM_152443.2	c.619A>G	p.(Asn207Asp)	3.68	23	Probably damaging (0.95)	Deleterious (0.00)	Absent
RDH5	NM_001199771.1	c.758T>G	p.(Met253Arg)	4.40	91	Probably damaging (0.98)	Deleterious (0.05)	Absent

Table S1. *Cont.*

Gene	RefSeq Id	Nucleotide variant	Protein variant	PhyloP	Grantham distance	PolyPhen	SIFT	EVS
RHO	NM_000539.3	c.448G>A	p.(Glu150Lys)	4.24	56	Probably damaging (0.99)	Deleterious (0.00)	Absent
RLBP1	NM_000326.4	c.346G>C	p.(Gly116Arg)	5.45	125	Probably damaging (1.00)	Tolerated (0.18)	Absent
RPE65	NM_000329.2	c.131G>A	p.(Arg44Gln)	5.77	43	Probably damaging (1.00)	Deleterious (0.02)	Absent
RPE65	NM_000329.2	c.751G>T	p.(Val251Phe)	6.26	50	Probably damaging (1.00)	Deleterious (0.03)	Absent
RPGRIP1	NM_020366.3	c.2480G>T	p.(Arg827Leu)	0.85	102	Probably damaging (0.99)	Deleterious (0.02)	Absent
SEMA4A	NM_022367.3	c.1033G>C	p.(Asp345His)	2.14	81	Probably damaging (0.99)	Deleterious (0.01)	Absent
SEMA4A	NM_022367.3	c.1049T>G	p.(Phe350Cys)	4.24	205	Probably damaging (1.00)	Deleterious (0.03)	Absent
TULP1	NM_003322.3	c.1138A>G	p.(Thr380Ala)	2.87	58	Benign (0.23)	Deleterious (0.00)	Absent
TULP1	NM_003322.3	c.1445G>A	p.(Arg482Gln)	6.10	43	Probably damaging (1.00)	Deleterious (0.00)	Absent
TULP1	NM_003322.3	c.1466A>G	p.(Lys489Arg)	4.97	26	Probably damaging (0.99)	Deleterious (0.00)	Absent
ZNF513	NM_144631.5	c.1015T>C	p.(Cys339Arg)	1.98	180	Probably damaging (0.99)	Deleterious (0.00)	Absent

EVS, exome variant server; PolyPhen, polymorphism phenotyping; SIFT, sorting tolerant from intolerant.

Table S2. *In silico* analysis of variants identified in Pakistani individuals with syndromic retinal dystrophies and their frequencies in normal individuals.

Gene	RefSeq Id	Nucleotide variant	Protein variant	PhyloP	Grantham distance	Polyphen	SIFT	EVS
ARL6	NM_032146.3	c.281T>C	p.(Ile94Thr)	4.56	89	Probably damaging (0.97)	Deleterious (0.01)	Absent
ARL13B	NM_182896.2	c.236G>A	p.(Arg79Gln)	6.34	43	Probably damaging (1.00)	Tolerated (0.16)	Absent
BBS1	NM_02464.9.4	c.442G>A	p.(Asp148Asn)	3.51	23	Probably damaging (1.00)	Tolerated (0.50)	Absent
BBS5	NM_152384.2	c.2T>A	p.(Met1Lys)	1.82	95	Probably damaging (1.00)	Deleterious (0.00)	Absent
BBS12	NM_152618.2	c.1589T>C	p.(Leu530Pro)	4.73	98	Probably damaging (1.00)	Deleterious (0.00)	Absent
CDH23	NM_022124.5	c.7198C>T	p.(Pro2400Ser)	6.10	74	Probably damaging (1.00)	Deleterious (0.00)	Absent
CDH23	NM_022124.5	c.8150A>G	p.(Asp2717Gly)	4.89	94	Probably damaging (1.00)	Deleterious (0.00)	Absent
TMEM67	NM_153704.5	c.1127A>C	p.(Gln376Pro)	4.24	76	Probably damaging (0.99)	Tolerated (0.15)	Absent

EVS, exome variant server; PolyPhen, polymorphism phenotyping; SIFT, sorting tolerant from intolerant.
